# Molecular evolution of the cytochrome c oxidase subunit *5*A gene in primates

**DOI:** 10.1186/1471-2148-8-8

**Published:** 2008-01-15

**Authors:** Monica Uddin, Juan C Opazo, Derek E Wildman, Chet C Sherwood, Patrick R Hof, Morris Goodman, Lawrence I Grossman

**Affiliations:** 1 Center for Molecular Medicine and Genetics, Wayne State University School of Medicine, Detroit MI 48201, USA; 2 Instituto de Ecología y Evolución, Universidad Austral de Chile, Casilla 567, Valdivia, Chile; 3 Perinatology Research Branch, National Institute of Child Health and Development, National Institutes of Health, Bethesda, MD 20892, USA; 4 Department of Obstetrics and Gynecology, Wayne State University School of Medicine and Hutzel Women's Hospital, Detroit, MI 48201, USA; 5 Department of Anthropology, The George Washington University, Washington DC 20052, USA; 6 Department of Neuroscience, Mount Sinai School of Medicine, New York, NY 10029, USA; 7 Department of Anatomy and Cell Biology, Wayne State University School of Medicine, Detroit MI 48201, USA

## Abstract

**Background:**

Many electron transport chain (ETC) genes show accelerated rates of nonsynonymous nucleotide substitutions in anthropoid primate lineages, yet in non-anthropoid lineages the ETC proteins are typically highly conserved. Here, we test the hypothesis that *COX5A*, the ETC gene that encodes cytochrome *c *oxidase subunit 5A, shows a pattern of anthropoid-specific adaptive evolution, and investigate the distribution of this protein in catarrhine brains.

**Results:**

In a dataset comprising 29 vertebrate taxa, including representatives from all major groups of primates, there is nearly 100% conservation of the *COX5A *amino acid sequence among extant, non-anthropoid placental mammals. The most recent common ancestor of these species lived about 100 million years (MY) ago. In contrast, anthropoid primates show markedly elevated rates of nonsynonymous evolution. In particular, branch site tests identify five positively selected codons in anthropoids, and ancestral reconstructions infer that substitutions in these codons occurred predominantly on stem lineages (anthropoid, ape and New World monkey) and on the human terminal branch. Examination of catarrhine brain samples by immunohistochemistry characterizes for the first time COX5A protein distribution in the primate neocortex, and suggests that the protein is most abundant in the mitochondria of large-size projection neurons. Real time quantitative PCR supports previous microarray results showing *COX5A *is expressed in cerebral cortical tissue at a higher level in human than in chimpanzee or gorilla.

**Conclusion:**

Taken together, these results suggest that both protein structural and gene regulatory changes contributed to *COX5A *evolution during humankind's ancestry. Furthermore, these findings are consistent with the hypothesis that adaptations in ETC genes contributed to the emergence of the energetically expensive anthropoid neocortex.

## Background

The electron transport chain (ETC) genes encode essential components of the mitochondrial machinery that carries out aerobic energy production. Since the integrity of this machinery must be maintained to sustain life, the mammalian ETC proteins are generally evolutionarily conserved. Among primates, however, the ETC genes show marked upsurges in the rate of nonsynonymous (amino-acid changing) substitutions [1-17]. These upsurges, indicative of adaptive evolution, have not occurred randomly; rather, they have occurred within anthropoid primates [9], a clade that includes New and Old World monkeys and apes, including humans. Life history and morphological features characteristic of this clade include a relatively enlarged cerebral cortex, prolonged intrauterine fetal development followed postnatally by prolonged dependency of the young on nurturing adults, and longer lifespan. The aerobic demand of these features makes it plausible that they have acted as selective pressures during anthropoid evolution, producing adaptive amino acid and possibly regulatory changes in aerobic energy producing ETC genes.

Previous work suggested that among ETC genes, *COX5A*, the nuclear gene encoding cytochrome *c *oxidase subunit 5A, may provide a striking example of adaptive evolution. Initial characterization of subunit 5A in human revealed a high degree of conservation with the corresponding subunit in cow [18]. In a subsequent bioinformatics study, Schmidt et al. [13] found that, among COX5A protein (COX5Ap) sequences from mammals, the human orthologue (the only primate representative in that dataset) differed by five amino acid residues from mouse, rat, cow and pig, all of which had identical amino acid sequences. Here, with newly generated *COX5A *coding region nucleotide sequences and bioinformatically extracted orthologues from publicly available whole genome sequences, we tested whether this ETC gene shows the pattern of anthropoid-specific adaptive evolution observed in 14 other ETC proteins [9,10]. We did so within the context of a robust statistical framework, employing the PAML package [19] to determine: 1) whether the anthropoid clade has a different ratio of nonsynonymous/synonymous substitution rates (i.e., omega value) when compared to other placental mammals and 2) whether particular sites in COX5Ap show the signature of positive selection within the anthropoid clade.

In addition to these sequence-based analyses, we investigated patterns of *COX5A *expression and localization in catarrhine brains using immunohistochemistry and quantitative real time PCR (qRT-PCR) techniques. Finally, we identified the anthropoid lineages on which changes in COX5Ap residues that interact with other cytochrome *c *oxidase (COX) subunits occurred. Based on these results, we explored the hypothesis that *COX5A *evolution in humankind's ancestry involved both protein structural and gene regulatory changes, and that the anthropoid-specific evolution observed in ETC proteins represents an adaptive response to the increased energy demands of an enlarged neocortex.

## Results and Discussion

**Figure 1:**
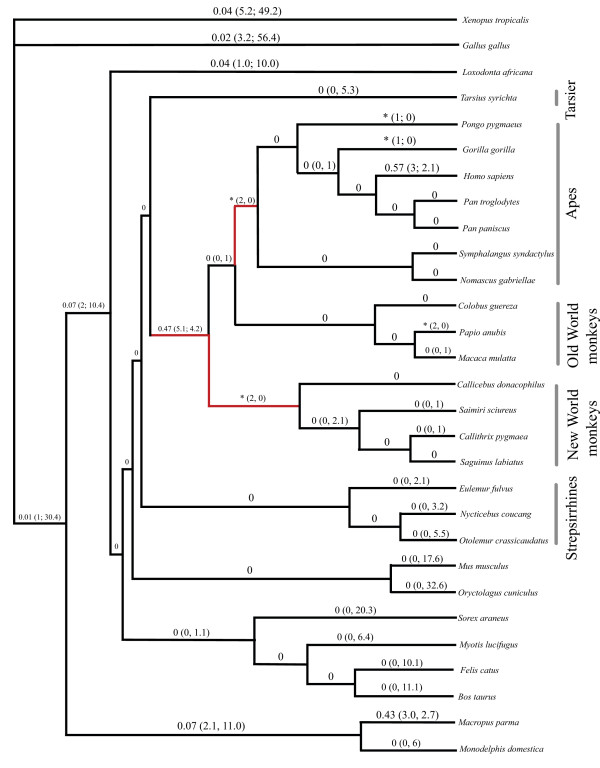
Phylogenetic tree depicting the omega ratios and (number of nonsynonymous and synonymous substitutions) for *COX5A *in each branch of the tree, as estimated by PAML under the free ratio (M1) model. "0" and "*" indicate, respectively, lineages on which the number of nonsynonymous and synonymous changes, as well as dN and dS, were estimated to be effectively 0 (i.e., < 0.00004); and lineages on which the number of synonymous changes was estimated to be 0 (i.e., omega is undefined). The anthropoid, New World monkey and ape stems, indicated in red, depict a classic pattern of positive selection [22]: markedly increased dN relative to dS followed by descendant lineages with a markedly decreased dN relative to ds.

**Figure 2:**
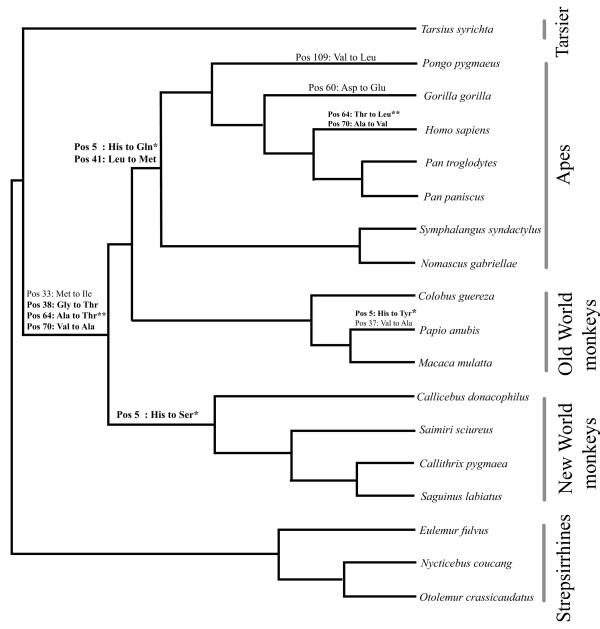
Phylogenetic tree depicting amino acid replacements inferred in COX5Ap within the anthropoid clade determined via maximum parsimony (ACCTRAN and DELTRAN) as implemented in PAUP* [51], and the codon substitution model [52] implemented in PAML 3.15. Non-prosimian outgroup species are not shown. Bold type indicates positively selected amino acids identified via the branch site test using PAML (Table 1). * Amino acid replacements in which the charge has changed, ** amino acid replacements in which the polarity has changed.

Complete *COX5A *nucleotide coding region and deduced amino acid sequences encoding the mature peptide were analysed in 26 vertebrate species, including newly generated data from 14 primate species: common chimpanzee (*Pan troglodytes*), bonobo (*Pan paniscus*), gorilla (*Gorilla gorilla*), orangutan (*Pongo pygmaeus*), red-cheeked gibbon (*Nomascus gabriellae*), siamang (*Symphalangus syndactylus*), mantled guereza (*Colobus guereza*), olive baboon (*Papio anubis*), pygmy marmoset (*Callithrix pygmaea*), white-lipped tamarin (*Saguinus labiatus*), white-eared titi monkey (*Callicebus donacophilus*), slow loris (*Nycticebus coucang*), brown greater galago (*Otolemur crassicaudatus*) and brown lemur (*Eulemur fulvus*); and one marsupial, the parma wallaby (*Macropus parma*). Partial sequences were obtained for three additional taxa (cat, rabbit, and chicken). Apart from a single amino acid difference in the elephant sequence, the protein sequence alignment showed no amino acid changes from the last common ancestor of placental mammals to present-day non-anthropoids, in a sampling that includes representatives from every major placental mammalian clade except Xenarthra (Fig. 1). Interestingly, the elephant is similar to anthropoids in that both share the aerobically demanding feature of lengthy gestation. Within anthropoids, 13 amino acid replacements were inferred by all ancestral state reconstruction methods, and eight of these occurred on the human lineage from the last common ancestor (LCA) of haplorhines (tarsier and anthropoids) (Fig. 2).

### Evidence for adaptive evolution

Results from the model-based codeml analyses confirm that *COX5A *omega ratios vary among lineages in vertebrates. Specifically, the statistical model assuming one omega value for all branches (i.e., the one ratio model) had a likelihood value of -1431.66, with an omega value of 0.048. The model that assumes an independent omega value for each branch (i.e., the free ratio model) had a likelihood value of -1388. According to the likelihood ratio test, the free ratio model fit the data significantly better than the one ratio model (p < 0.01). The inferred omega values and the numbers of nonsynonymous and synonymous substitutions under the free ratio model are presented in Fig. 1. In addition, the model that segregates anthropoids from non-anthropoid placental mammals fit the data significantly better than the model that assigned the same omega value to all placental mammals (p < 10^-5^), indicating that the ratio of nonsynonymous/synonymous substitution rates differs in this clade; in particular, the omega value inferred for anthropoids is 52 times greater than that inferred for non-anthropoid placental mammals (0.431 vs. 0.008; Table 1).

**Table 1:** Parameter estimates and likelihood values under branch and branch-site models.

Model	P	likelihood	Parameters estimated	Positively selected sites
*Branch Models*				
1ω	1	-1431.66	ω all branches = 0.048	N/A
2ω	2	-1431.41	ω placental mammals = 0.054; background = 0.04	N/A
3ω	3	-1408.70	ω anthropoid = 0.431; ω other placentals = 0.008; ω background = 0.042	N/A
Free Ratio	55	-1388.00	ω estimated independently for each branch (see Fig. 1)	N/A
*Branch-site models*				
Model A	4	-1399.23	p_0 _= 0.90; p_1 _= 0.012; p_(2a+2b) _= 0.085; ω_0 _= 0.021; ω_1 _= 1; *background *ω_2a _= 0.021; ω_2b _= 1; *foreground*, ω_2a _= ω_2b _= 4.61	**5 (1.00); 38 (0.99); **41 (0.53); **64 (1.00); 70 (0.97)**
Model A (omega fixed)	3	-1403.73	p_0 _= 0.80; p_1 _= 0.011; p_(2a+2b) _= 0.192; ω_0 _= 0.02; ω_1 _= 1; *background *ω_2a _= 0.02; ω_2b _= 1; *foreground*, ω_2a _= ω_2b _= 1	Not allowed

P, number of parameters. Numbers in parentheses represent the posterior probability of each amino acid listed to be in the class of sites under positive selection.

Despite this elevated ratio in anthropoids, we note that the overall ratio is still less than one. This result, however, is based on the assumption that all codons are under the same selective pressure and thus share the same underlying omega value. Because this assumption is "grossly unrealistic" [20], and because important changes in function can result from a few key amino acid changes that do not elevate the overall omega ratio above one [21], we conducted a branch-site test of adaptive evolution. Here, the Anthropoidea as a total group was fixed as the foreground lineage, i.e., the branches on which there was permitted a class of sites with an omega > 1. Results from this test provide evidence of positive selection on particular sites during anthropoid evolution: the model allowing a class of sites with an omega higher than one (i.e., positive selection) fit the data significantly better than an alternative model that fixed omega at one for this same site class (p < 0.002). Specifically, five sites were identified as positively selected, four of which had a posterior probability > 0.95 (Table 1). Furthermore, we note that despite the many amino acid replacements inferred to have occurred throughout the Anthropoidea (see below), the five positively selected sites show changes occurring predominantly on stem lineages (anthropoid, ape, and New World monkey) and on the human terminal branch. The localization of these particular sites to these three stem lineages, combined with the M1 analysis (Fig. 1) depicting a classic sequence [22] of markedly increased dN relative to dS, followed by descendant lineages with a markedly decreased dN relative to dS, presents a pattern strongly indicative of positive selection.

Ancestral state reconstruction methods confirm and extend results obtained by the codeml analysis. Among the five sites identified as positively selected by the branch site test, four were inferred to have changed on more than one anthropoid lineage, and three were inferred to have changed on the anthropoid stem (Fig. 2). In addition, all three ancestral state reconstruction methods inferred a fourth amino acid change on the anthropoid stem that was not identified by the branch site test: a methionine to isoleucine at residue 33. It is tempting to speculate that this replacement may have acted as a "permissive" change that then allowed the selection of the other three amino acid changes occurring on this and perhaps other descendant anthropoid branch(es). This sequence of events was recently demonstrated in an elegant study by Ortlund et al. [23] in their investigation of cortisol-sensitive glucocorticoid receptor (GR) evolution in vertebrates. Using a combination of structural, phylogenetic and functional analyses of ancestral corticosteroid receptors, the authors identified a small set of permissive amino acid changes that stabilized specific elements of the protein. In conjunction with additional changes that diminished the receptor's sensitivity to aldosterone and increased its sensitivity to cortisol, these permissive changes allowed an additional and subsequent set of function-switching amino acid changes that conferred a fully GR-like phenotype that is sensitive only to cortisol. With respect to COX5Ap, verification of this hypothesis in future studies would require additional, fine-tuned functional data regarding the effects of each of the observed replacements alone and in concert on the activity of the reconstructed protein.

### Expression and localization of COX5A

**Figure 3:**
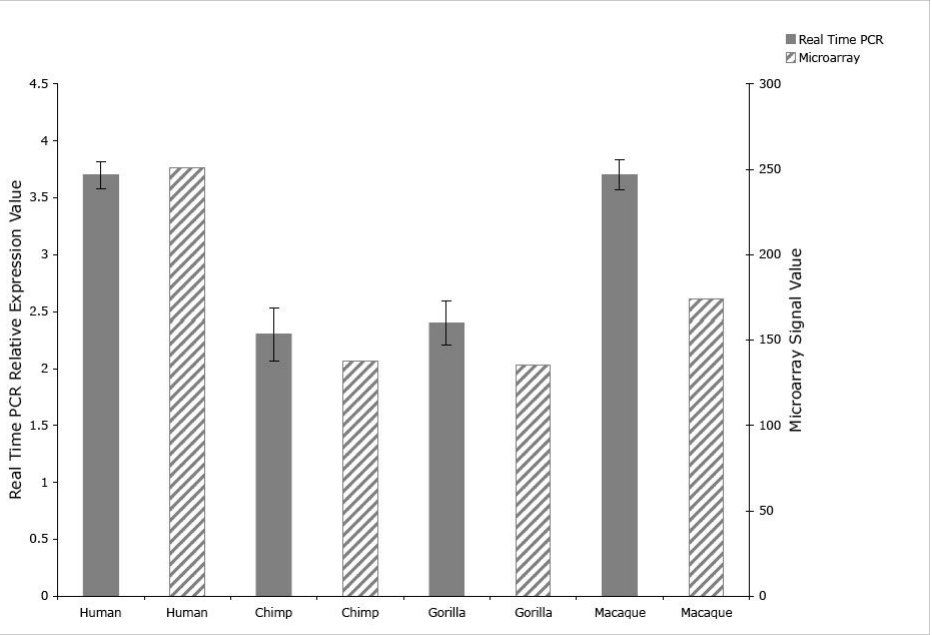
*COX5A *Expression levels as determined by quantitative RT-PCR (solid bars) and microarray signal values (hatched bars). qRT-PCR expression levels are expressed in relative terms, with samples compared to a calibrator amplified from human reference total RNA and represented as species means (± SEM). Samples were tested in triplicate, providing 9 total measurements for human and macaque and three total measurements for chimpanzee and gorilla. Microarray expression levels were as determined in [24].

Quantitative RT-PCR results showed that relative *COX5A *gene expression levels in the anterior cingulate cortex vary among human, chimpanzee, gorilla, and macaque (Fig. 3). Specifically, human and macaque showed similar gene expression levels, as did chimpanzee and gorilla, and the first pair of species showed higher levels of expression than the second pair (Fig. 3). This pattern confirms the trend previously suggested by microarray analysis [24], and represents a more robust dataset from which to make such observations: unlike the previous experiment, which involved hybridizing non-human samples to a human-specific microarray, these qRT-PCR results are not confounded by sequence mismatches between sample and microarray chip. Specifically, the *COX5A *expression results reported by Uddin et al. [24] were based on an Affymetrix probe set (203663_s_at) that shows 0.8% difference with the published chimpanzee genome sequence [25] and 7.3% difference with the published rhesus macaque sequence [26]. Thus, the present results suggest that *COX5A *has undergone not only coding region sequence evolution, but also regulatory evolution in anthropoid primates. This hypothesis is supported by the observation of primate *COX5A *expression patterns in other tissues that differ from those reported here: in microarray experiments performed on fibroblast cell lineages, humans showed a lower *COX5A *expression level than did bonobos or gorillas, and comparison of the human and bonobo expression levels showed a significant difference [27]. Nevertheless, without additional samples from additional chimpanzees and gorillas, the possibility that our results are due to interindividual differences within these species and/or age-related differences in gene expression profiles cannot formally be ruled out.

**Figure 4:**
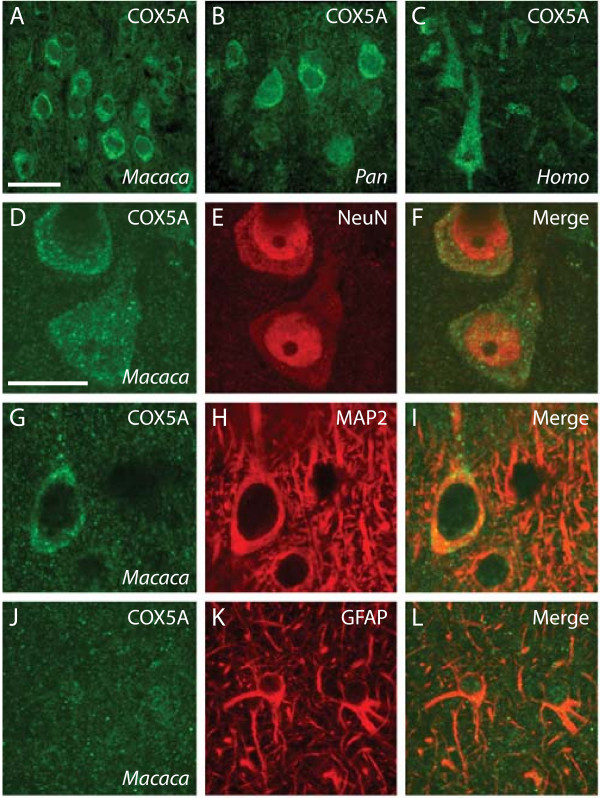
Immunohistochemical staining of COX5A protein in the dorsolateral prefrontal cortex. The distribution of staining using a monoclonal antibody against COX5Ap in macaque monkey (A), chimpanzee (B), and human (C). Panels D-L show double label immunostaining from macaque monkey. Images in A-I are taken from layer III. Images in J-L are taken from layer I. Scale bar in panel A = 50 μm applies to A-C. Scale bar in D = 25 μm applies to D-L.

Among macaques, gorillas, chimpanzees, and humans, the localization of COX5Ap as revealed by immunohistochemistry showed a punctate staining pattern that is consistent with the morphology of mitochondria (Fig. 4, data from gorilla not shown). Similar staining patterns were obtained in all species with both monoclonal and polyclonal antibodies. As evident by colocalization with microtubule-associated protein 2 (MAP2) and neuron-specific nuclear protein (NeuN), COX5Ap was particularly enriched in the cytoplasm of the soma and proximal apical dendrite of neurons, especially large pyramidal cells. We also observed diffuse COX5Ap-immunoreactive puncta throughout the neuropil, which corresponds to the space occupied by glia, dendritic processes, axons, and synapses. Double immunostaining against glial fibrillary acidic protein (GFAP) demonstrated that astrocytes do not show high levels of COX5Ap. Indeed, 95.3% of cells that are enriched with COX5Ap also contain the neuron-specific protein NeuN in macaques, as estimated by optical disector counting. The white matter did not display a high density of COX5Ap staining.

These results suggest that COX5Ap is most abundant in the mitochondria of large-size projection neurons of the neocortex. It is noteworthy that neocortical enlargement in primates is associated with increasing numbers of large pyramidal neurons that are intensely neurofilament H protein-immunoreactive, have long-range projecting axons, and are presumably metabolically costly [28]. To understand more completely the functional role of *COX5A *in neocortical circuits and the possible consequences of positive selection on this gene, it will be necessary to define further the phenotype and distribution of cells in the brain that show high levels of expression for COX5Ap using strategies such as laser microcapture dissection combined with qRT-PCR or mass spectroscopy proteomics in future studies.

### Role of accelerated COX evolution

As we have demonstrated, changes in COX5Ap are rare among placental mammals outside the primate clade. Among four positively selected sites identified by the branch site test with posterior probabilities > 0.95, two showed changes in their physicochemical properties: residue 5, which changed from positively charged to neutral on the ape and New World monkey stems and on the baboon terminal (Fig. 2); and residue 64, which changed from nonpolar to polar in the anthropoid stem and from polar to nonpolar on the human terminal. Interestingly, the charge neutralizations are parallel to those observed at the binding site for cytochrome *c *on COX in anthropoids, where the binding site changes reduced the electrostatic interaction between the docked molecules [15]. Of note, one amino acid change on the human terminal at position 70 (Ala to Val) is a reversal of a change on the anthropoid stem (Fig. 2).

Although the role of the inferred amino acid changes in COX5Ap is currently unknown, it is noteworthy that the changes in this protein are located in residues that are in proximity to other, physically close, COX subunits (Table 2). Of the five positively selected sites identified by the branch site test in COX5Ap, seven replacements within 10 Å of these sites have occurred in COX2p and COX4p during anthropoid evolution. Examination of the nature of the changes suggests there has been an electrostatic change such that the interaction between position 5 of COX5Ap and residues 52 and 57 of COX2p has been neutralized; in addition, residues 55 and 56 of this subunit have changed from polar to nonpolar. Furthermore, the potentially interacting, altered residues of COX4p (22 and 63) have changed from neutral to positive charge (Fig. 2 and Table 2), forming a positive patch. Of particular interest is that COX4p residue 22, which has changed from Tyr to His, is adjacent to the proposed ATP binding site that includes COX4p 20 [29].

**Table 2:** Inferred amino acid replacements in COX2p and COX4p that occur in proximity to positively selected sites in COX5Ap among anthropoid primate stem lineages.

COX5Ap		COX2p		COX4p	
Residue	Lineage	Residue	Lineage	Residue	Lineage

5 (H to Q)	Ape stem	52 (H to N)	Catarrhine* stem		
(H to S)	New World monkey stem	54 (S to N)	Catarrhine stem		
		55 (T to I)	Catarrhine stem		
		56 (M to T)	Catarrhine stem		
		56 (T to S)	Large-bodied ape stem		
		57 (D to N)	New World monkey stem		
38 (G to T)	Anthropoid stem	52 (H to N)	Catarrhine stem		
		54 (S to N)	Catarrhine stem		
		55 (T to I)	Catarrhine stem	22 (Y to H)	Ape stem
		56 (M to T)	Catarrhine stem		
		56 (T to S)	Large-bodied ape stem		
41 (L to M)	Ape stem	52 (H to N)	Catarrhine stem		
		54 (S to N)	Catarrhine stem		
64 (A to T)	Anthropoid stem			63 (Q to K)	Catarrhine stem
70 (V to A)	Anthropoid stem			22 (Y to H)	Ape stem

Interaction is taken as a distance < 10 Å between residues.

*Catarrhines include Old World monkeys and apes, including humans

An additional consequence of the location of COX5Ap in close contact to the nucleotide-binding domain of COX4p is potential regulation by 3,5-diiodothyronine (T_2_), a thyroid hormone produced when the iodothyronine deiodinase D3 acts over thyroxine (T_4_) [30]. T_2 _has biological activity stimulating mitochondrial respiration by nuclear independent pathways [30,31] and is known to bind to COX5Ap and abolish the allosteric inhibition of respiration at high ATP/ADP ratios [32]. More generally, triiodothyronine (T_3_) thyroid hormone is considered to be a major regulator of mitochondrial activity [33] and has been shown to regulate the expression of a number of COX subunits, inducing functional increases in COX enzyme activity [34]. *COX5A *amino acid evolution may thus represent the product of selective pressures acting on the regulation of metabolism. In the context of this hypothesis, we note that thyroid hormones are known to play a critical role in early brain development [35-37] and that humans show evidence for a greater affinity of transthyretin and/or thyroxine-binding globulin for thyroid hormone when compared to chimpanzees [38]. Thus, it is plausible that the interaction of thyroid hormones with COX5Ap and other COX subunits may affect both the regulation of metabolism and the growth and development of important organ systems.

## Conclusion

We have shown that *COX5A *has been subject to protein structural evolutionary changes in anthropoid primates and that these changes have likely been accompanied by additional gene regulatory changes affecting *COX5A *expression levels. Furthermore, we have suggested that these changes could affect aerobic energy metabolism among members of this group, in part because COX5Ap is a target of the thyroid hormone T_2 _[32]. In addition, a recent analysis of cellular scaling in the neocortex of anthropoids showed that elevated glia-neuron ratios correlate with brain enlargement [39], pointing to the role of glia in providing metabolic support to neurons requiring greater energy consumption for function. Because the anthropoid amino acid changes are located in the mitochondrial matrix, close to coevolving sites in subunits 2 and 4 (Table 2) proposed to have a COX regulatory function [29], the COX5Ap amino acid replacements observed in this study may have promoted better energy regulation in an increasingly high demand environment. While alternative interpretations of the data are possible – that, for instance, the higher rate of nonsynonymous substitutions in *COX5A *in anthropoids may be a function of their larger body and smaller presumed effective population size [40], which would reduce the effectiveness of purifying selection on slightly deleterious amino acid replacements [41]; or that the changed composition of mtDNA in anthropoids [42] may be the "motor" driving the putative positive selection of nuclear-encoded ETC [42], we note that COX5Ap amino acid sequences are identical in mammals with a wide range of body sizes and basal metabolic rates (e.g., shrew, cow), and that, within the context of intergenomic interactions between nuclear- and mitochondrially encoded ETC genes, it has been shown that rapid evolution in mitochondrially encoded subunits can indeed be associated with rapid divergence in interacting subunits [43] and that positive selection is particularly pronounced among interacting residues in ETC proteins [15,44]. More broadly, given that some of the main phenotypic differences associated with anthropoid origins are related to aerobic energy metabolism (e.g., longer lifespan, larger neocortex, and prolonged fetal development), both structural and regulatory evolutionary change in electron transport chain genes are likely to have contributed to the emergence and evolution of anthropoid primates [8,9,24,45].

## Methods

### DNA sequences

We sequenced the COX5A coding region for 16 mammalian species, including eight apes: human (*Homo sapiens *(2X)), common chimpanzee (*Pan troglodytes*), bonobo (*Pan paniscus*), gorilla (*Gorilla gorilla*), orangutan (*Pongo pygmaeus*), red-cheeked gibbon (*Nomascus gabriellae*) and siamang (*Symphalangus syndactylus*); two Old World monkeys: mantled guereza (*Colobus guereza*) and olive baboon (*Papio anubis*); three New World monkeys: pygmy marmoset (*Callithrix pygmaea*), white-lipped tamarin (*Saguinus labiatus*) and white-eared titi monkey (*Callicebus donacophilus*); three strepsirrhines: slow loris (*Nycticebus coucang*), brown greater galago (*Otolemur crassicaudatus*) and brown lemur (*Eulemur fulvus*) and one marsupial, the parma wallaby (*Macropus parma*) via RACE-PCR [33]. Four additional primate and two outgroup sequences were obtained from public databases: Philipine tarsier (*Tarsius syrichta*, AY_236506), common squirrel monkey (*Saimiri sciureus*, AY_585857), rhesus monkey (*Macaca mulatta*, AY_585861), human (NM_004255), mouse (*Mus musculus*, NM_007747), and cow (*Bos Taurus*, NM_00100289). Finally, 8 additional outgroup species were obtained from the ENSEMBL public database, including: frog (*Xenopus tropicalis*, ENSXETT00000045679), chicken (*Gallus gallus*, ENSGALT00000022855), gray short-tailed opossum (*Monodelphis domestica*, ENSMODT00000011691), African elephant (*Loxodonta africana*, ENSLAFT00000006266), cat (*Felis catus*, ENSFCAT00000010590), little brown bat (*Myotis lucifugus*, ENSMLUT00000013702), shrew (*Sorex araneus*, ENSSART00000003977) and rabbit (*Oryctolagus cuniculus*, ENSOCUT00000003256). Species in which there was more than one copy of *COX5A*, or for which poor reads were available, were not included in the study. All sequences newly obtained in this study were deposited in GenBank (DQ987236–DQ987252). Human reference sequence NM_004255 was used in the sequence analyses described below.

RNA was isolated either with the phenol-chloroform method or the RNeasy^® ^midi kit (Qiagen, Valencia, CA). RACE PCR [33] was performed on these RNAs using the following primers: Pri_R1, 5' GGTATCATCAGTATTTCCAGG 3'; Pri_R2, 5' TTAAGTTGGTCTAAGTTCCTGG 3'; Pri_R3, 5' CTTATGAGGTCCTGCTTTGTC 3'; Apes_R1, 5' TTTGTCAAGGCCCAGTTCCTC 3'; Apes_R2, 5' GAGTGGAGATTCCCAGTTCATT 3'; Apes_R3, 5' TTGGTCTAAGTTCCTGGATGAC 3'; 5AF1, 5' CATGGGTCACANGAGCANGA 3'; 5AF2, 5' GAGTTTGATGCTCGCTGGGT 3'; 5AF3, 5' GCCAGAYATNGATGCYTGGGA 3'. RACE PCR conditions were: 30 cycles of denaturation, 94°C, 40 s; annealing, 57°C, 30 s; and extension 72°C, 1 min and 10 s; initial denaturation at 94°C for 2 min and a final extension at 72°C for 7 min. RACE PCR products were visualized on 1% agarose gels and products were extracted using the Qiaquick^® ^(Qiagen, Valencia, CA) gel extraction kit. DNA sequencing reactions were performed using the Big Dye^® ^(Applied Biosystems, Foster City, CA) cycle sequencing kit according to the manufacturer's specifications.

### Sequence analyses

Phylogenetic relationships used to investigate patterns of *COX5A *nucleotide and amino acid sequence evolution were obtained from the literature [46,47]. Sequences were aligned using ClustalX 1.83.1 [48]. The PAML 3.15 [19] package was used to investigate the signature of positive selection among specific lineages and sites in the phylogeny. First, to investigate if rate heterogeneity existed among lineages, we compared a one omega (dN/dS) model (M0), which assumes the same relative rate (nonsynonymous divided by synonymous rate) for all lineages, with the free ratio model (M1), which allows rates to vary freely among the branches. Second, to investigate if the omega ratio varied among particular branches of interest in the phylogeny, we compared two different branch models. In the first model, two omega ratios were specified, one for all placental mammals (as a total group) and another for the remaining branches in the tree (i.e., the background). In the second model, three omega ratios were specified, one for the anthropoid clade (as a total group), another for the non-anthropoid placental mammals, and another for the remaining branches in the tree (i.e., the background). Finally, because all of these models assume a homogeneous omega ratio across codons, we also applied a branch-site test of positive selection on the anthropoid clade [49,50]. Model A (branch-site test of positive selection) assumes four classes of sites, the first two classes having omega values 0 < Δ_0 _< 1 and Δ_1 _= 1, assuming purifying selection and neutrality, respectively. The other two classes have the same assumption for the background branches, but allow for positive selection (i.e. Δ_2_>1) on the foreground branches. This model was compared with the same model A, but fixing the last two site classes to 1 according to Zhang et al. [50]. In all cases we checked for the existence of multiple local optima, running all models three times, with three different starting omega values (0.5, 1, and 2). Models were compared using the likelihood ratio test.

In addition to the previous model-based analyses, we reconstructed ancestral *COX5A *coding region nucleotide sequences to infer the amino acid changes that occurred among the sampled vertebrate lineages. Sequences were reconstructed, translated, and compared by three different methods: a maximum parsimony framework using ACCTRAN and DELTRAN algorithms as implemented in PAUP* [51], and the codon substitution model [52] implemented in PAML 3.15 [19]. Finally, to identify potential interactants with the COX5Ap amino acid replacements that occurred during anthropoid evolution, coding region nucleotide sequences were reconstructed, translated and compared for COX2p and COX4p (i.e., the genes encoding COX subunits in closest proximity to COX5Ap) using a maximum parsimony framework (ACCTRAN and DELTRAN). Ancestral reconstruction of COX4p sequences were based on analyses of sequences from Wildman et al. [16]. Ancestral reconstruction of COX2p sequences were based on analyses of sequences listed in Additional file 1. Proximity of potentially interacting residues in COX2p and COX4p was determined based on the crystal structure of cytochrome *c *oxidase 2oCC, using Swiss-PDB viewer to identify the replacements occurring at a distance of 10 Å or less.

### Real time PCR

To examine and potentially validate the pattern of *COX5A *upregulation in humans reported in Uddin et al. [24], a SYBR green-based quantitative real-time quantitative PCR (qRT-PCR) strategy was designed to test the same individuals and species originally investigated by the microarray experiments: *Homo sapiens *(N = 3), *Pan troglodytes *(N = 1), *Gorilla gorilla *(N = 1) and *Macaca mulatta *(N = 3). cDNA was synthesized using total RNA (50 ng) obtained from the anterior cingulate cortex of each individual using the Stratascript^® ^First-Strand Synthesis System from Stratagene (La Jolla, CA) by following manufacturer's protocols. A serial dilution of reference cDNA was also amplified for use in standard curve construction and as a calibrator for subsequent real-time experiments using Stratagene's qRT-PCR Human Reference Total RNA (100 ng). *COX5A*-specific primers were designed in regions that were 100% conserved among the species and individuals hybridized to the microarray: COX5AF2 (5'-ATCCAGTCAGTTCGCTGCTA-3') and COX5AR2 (5'GTTTATCCCTTTACGCAATTCC-3') amplified a 123-bp product. *COX5A *expression levels were scaled to that of *EIF3S7*, a control gene on the Affymetrix HG-U133A and HG-U133B microarray chips that demonstrated one of the lowest standard deviations in expression levels among all tested samples (data not shown). Primers were designed in regions that were 100% conserved among species with available genomic data: *EIF3F1 *(5'-AATGTGTTTGCCACTGATGC-3') and *EIF3R1 *(5'AGGAGGTCAAAGTCAGAGTTGTC-3') amplified a 137-bp product. For both primer sets, one primer was designed to fall in two exons in order to reduce the possibility of amplifying products from potentially contaminating genomic DNA. All primers were HPLC purified, and optimal concentrations were established using serial dilutions of reference cDNA in preliminary experiments.

Standard curves for both primer sets produced efficiencies > 91% and Rsq > 0.99, thus meeting or exceeding the manufacturer's recommended values for ensuring a robust qRT-PCR assay. Quantitative RT-PCR reactions were run on Stratagene's Mx3000P instrument in triplicate for each and primer set on each individual and on each no template control (NTC) using the FullVelocity^® ^SYBR^® ^Green QPCR Master Mix^® ^(Stratagene) following manufacturer's protocols and including both the optional diluted reference dye and 0.25 Δl of AmpErase^® ^Uracil *N*-glycosylase from Applied Biosystems (Foster City, CA). After ensuring that all NTCs were negative and that all samples fell within the linear range of amplification as previously determined by the standard curves, relative *COX5A *expression levels were calculated using the 2^-ΔΔC ^T method described in Livak and Schmittgen [53] for target and reference samples, amplified in separate wells.

### Immunohistochemistry

The localization of COX5Ap was examined in neocortex samples from non-geriatric adult macaque monkeys (*Macaca maura*), gorillas, common chimpanzees, and humans (N = 2 individuals per species). Macaque monkey brains were obtained from animals transcardially perfused with 4% paraformaldehyde as part of an unrelated study. Brains of gorillas, chimpanzees, and humans were collected within 14 hours of death and immersed in 10% formalin. Tissue sections were cut at a thickness of 40 μm with a freezing microtome.

Immunohistochemistry was carried out on sections from the dorsolateral prefrontal cortex. Initial single-label immunohistochemical staining was performed using two different antibodies raised against the human COX5Ap, a mouse monoclonal IgG antibody (A-21363; Molecular Probes; dilution 1:200) and a rabbit polyclonal IgG antibody (11448-1-AP; Proteintech Group, Inc.; dilution 1:50). Prior to immunostaining, sections were pretreated for antigen retrieval by incubation in 10 mM sodium citrate buffer (pH 3.5) at 37°C in an oven for 30 minutes. Sections were then incubated in the primary antibody diluted in PBS with 2% normal serum and 0.1% Triton X-100 detergent for approximately 48 hours on a rotator at 4°C. After rinsing in PBS, sections were incubated in a 1:200 dilution of Alexa Fluor 488-conjugated anti-mouse or anti-rabbit secondary antibody. Sudan Black B (1% in 70% ethanol) was used to quench the autofluorescence generated by lipofuscin pigments.

To characterize further the cellular localization of COX5Ap, we also performed double-labeling immunofluorescence experiments in the perfusion-fixed macaque monkey samples using the polyclonal COX5Ap antibody in combination with mouse monoclonal antibodies against (1) microtubule-associated protein 2 (MAP2) (MAB3418, clone AP20; Chemicon International; dilution 1:200), (2) neuron-specific nuclear protein (NeuN) (MAB377; Chemicon International; dilution 1:100), and (3) glial fibrillary acidic protein (GFAP) (MU020-UC; BioGenex Laboratories, Inc.; dilution 1:10,000). Both primary antibodies were incubated with the tissue simultaneously for 48 hours as described above. Immunoreactivity was revealed using an Alexa Fluor 488-conjugated anti-rabbit secondary antibody to visualize COX5Ap and an Alexa Fluor 568-conjugated anti-mouse secondary antibody to visualize MAP2, NeuN, and GFAP. In all immunohistochemistry runs, specificity of the reaction was confirmed by processing negative control sections excluding the primary antibody. No immunostaining was observed in control sections.

Brain sections were imaged with a BioRad MRC 1024 confocal laser scanning system coupled to an Olympus IX-70 microscope. Images were acquired with a 40× objective lens, a slice thickness of 1.5 μm, and a *z *step of 0.5 μm. Figures show a *z *projection of three contiguous confocal slices.

Quantification of the percentage of COX5Ap-immunoreactive cells that also contained NeuN in macaques (n = 2) was performed using the optical disector method as described previously [39] implemented in StereoInvestigator software (MBF Bioscience, Williston, VT). In two sections for each monkey, optical disector frames (30 μm × 30 μm × 6 μm) were placed within a contour drawn around the entire cortical width in the dorsolateral prefrontal cortex (Brodmann's area 9). Optical disectors were placed within this contour in a systematic random fashion using the fractionator method. Counting frames were investigated with a 63× objective (Zeiss Plan-Apochromat, N.A. 1.4) and cells were marked if their nucleolus was contained within the three-dimensional limits of the counting frame. Our estimate of the percentage of COX5A+NeuN-immunoreactive cells is based on a total sample of 619 cells.

## Authors' contributions

JCO, MU, DEW, and CCS carried out the experimental work and data analysis, MG, and LIG carried out data analysis, and JCO, MU, DEW, CCS, PRH, MG, and LIG contributed to manuscript preparation and provided conceptual framework. All authors read and approved the final manuscript.

## Supplementary Material

Additional file 1 Species name and Accession number of COII sequences used in this study. The listed taxa and associated COII sequences are the data used to determine the ancestral reconstructions of COX2p sequences reported in the text and in Table 2. Click here for file
