# Supplementary material for: Molecular evolution of the cytochrome c oxidase subunit 5A gene in primates
Source: BMC Evol Biol. 2008 Jan 15;8:8. doi: 10.1186/1471-2148-8-8 (PMC2241769; doi:10.1186/1471-2148-8-8)
Supplement: Additional file 1 — Species name and Accession number of COII sequences used in this study. The listed taxa and associated COII sequences are the data used to determine the ancestral reconstructions of COX2p sequences reported in the text and in Table 2. [file 1471-2148-8-8-S1.doc]

| Additional File 1. Species name and Accession number of COII sequences used in this study. | | | |
| --- | --- | --- | --- |
| Species name | Order | Common name | Accession |
| Bos taurus | Cetartiodactyla | Cow | [AF493542](http://www.ncbi.nlm.nih.gov/entrez/viewer.fcgi?db=nuccore&val=20149081) |
| Mus musculus | Rodentia | Mouse | AY172335 |
| Galago senegalensis | Primates | Senegal Bushbaby | [M80905](http://www.ncbi.nlm.nih.gov/entrez/viewer.fcgi?db=nuccore&id=337104) |
| Daubentonia madagascariensis | Primates | Aye-aye | L22776 |
| Propithecus tattersalli | Primates | Golden-crowned Sifaka | L22782 |
| Cheirogaleus medius | Primates | Lesser Dwarf Lemur | L22775 |
| Varecia variegata | Primates | Black-and-white Ruffed Lemur | L22785 |
| Eulemur macaco | Primates | Black Lemur | AF081044 |
| Hapalemur griseus | Primates | Gray Bamboo Lemur | L22778 |
| Lemur catta | Primates | Ring-tailed Lemur | L22780 |
| Tarsius bancanus | Primates | Horsefield’s Tarsier | L22783 |
| Tarsius syrichta | Primates | Philippine Tarsier | L22784 |
| Lagothrix lagothricha | Primates | Brown Woolly Monkey | EF999916 |
| Alouatta palliata | Primates | Mantled Howler | AF216252 |
| Chlorocebus aethiops | Primates | Grivet | M58005 |
| Macaca fascicularis | Primates | Crab-eating Macaque | M58008 |
| Macaca mulatta | Primates | Rhesus Monkey | M74005 |
| Papio anubis | Primates | Olive Baboon | M74007 |
| Papio hamadryas | Primates | Hamadryas Baboon | M74008 |
| Theropithecus gelada | Primates | Gelada | M74009 |
| Mandrillus leucophaeus | Primates | Drill | M74006 |
| Cercocebus galeritus | Primates | Tana River Mangabey | M74004 |
| Symphalangus syndactylus | Primates | Siamang | M58007 |
| Pongo pygmaeus | Primates | Orangutan | D38115 |
| Gorilla gorilla | Primates | Gorilla | M58006 |
| Pan paniscus | Primates | Bonobo | D38116 |
| Pan troglodytes | Primates | Common Chimpanzee | [M58009](http://www.ncbi.nlm.nih.gov/entrez/viewer.fcgi?db=nucleotide&val=336513) |
| Homo sapiens | Primates | Human | [EU095539](http://www.ncbi.nlm.nih.gov/entrez/viewer.fcgi?db=nucleotide&val=156077355) |
